# Risk Factors and Outcomes of Candidemia Caused by Biofilm-Forming Isolates in a Tertiary Care Hospital

**DOI:** 10.1371/journal.pone.0033705

**Published:** 2012-03-30

**Authors:** Mario Tumbarello, Barbara Fiori, Enrico Maria Trecarichi, Patrizia Posteraro, Angela Raffaella Losito, Alessio De Luca, Maurizio Sanguinetti, Giovanni Fadda, Roberto Cauda, Brunella Posteraro

**Affiliations:** 1 Institute of Infectious Diseases, Università Cattolica del Sacro Cuore, Rome, Italy; 2 Institute of Microbiology, Università Cattolica del Sacro Cuore, Rome, Italy; 3 Laboratory of Clinical Pathology and Microbiology, Ospedale San Carlo, Rome, Italy; 4 Hospital Pharmacy, Università Cattolica del Sacro Cuore, Rome, Italy; 5 Institute of Hygiene, Università Cattolica del Sacro Cuore, Rome, Italy; The University of Texas at San Antonio, United States of America

## Abstract

**Background:**

Very few data exist on risk factors for developing biofilm-forming *Candida* bloodstream infection (CBSI) or on variables associated with the outcome of patients treated for this infection.

**Methods and Findings:**

We identified 207 patients with CBSI, from whom 84 biofilm-forming and 123 non biofilm-forming *Candida* isolates were recovered. A case-case-control study to identify risk factors and a cohort study to analyze outcomes were conducted. In addition, two sub-groups of case patients were analyzed after matching for age, sex, APACHE III score, and receipt of adequate antifungal therapy. Independent predictors of biofilm-forming CBSI were presence of central venous catheter (odds ratio [OR], 6.44; 95% confidence interval [95% CI], 3.21–12.92) or urinary catheter (OR, 2.40; 95% CI, 1.18–4.91), use of total parenteral nutrition (OR, 5.21; 95% CI, 2.59–10.48), and diabetes mellitus (OR, 4.47; 95% CI, 2.03–9.83). Hospital mortality, post-CBSI hospital length of stay (LOS) (calculated only among survivors), and costs of antifungal therapy were significantly greater among patients infected by biofilm-forming isolates than those infected by non-biofilm-forming isolates. Among biofilm-forming CBSI patients receiving adequate antifungal therapy, those treated with highly active anti-biofilm (HAAB) agents (e.g., caspofungin) had significantly shorter post-CBSI hospital LOS than those treated with non-HAAB antifungal agents (e.g., fluconazole); this difference was confirmed when this analysis was conducted only among survivors. After matching, all the outcomes were still favorable for patients with non-biofilm-forming CBSI. Furthermore, the biofilm-forming CBSI was significantly associated with a matched excess risk for hospital death of 1.77 compared to non-biofilm-forming CBSI.

**Conclusions:**

Our data show that biofilm growth by *Candida* has an adverse impact on clinical and economic outcomes of CBSI. Of note, better outcomes were seen for those CBSI patients who received HAAB antifungal therapy.

## Introduction

*Candida* bloodstream infections (CBSIs) are the fourth most common infections among hospitalized patients [1], accounting for 8% to 15% of hospital-acquired BSIs [2]. They are considered high-morbidity infections [3], [4], with significant hospital costs [5], [6], largely due to increased hospital length of stay (LOS) and costs for antifungal therapy [2]. The excess of hospital stay attributable to invasive *Candida* infections has been reported to range from 10 to 30 days in the United States [2], and it could have been underestimated because of the early mortality associated with delayed therapy [7], [8]. Recently, inappropriate antifungal treatment resulted in prolonged hospital LOS and increased hospital costs [9].

Candidemia is frequently associated with the biofilm growth of *Candida* organisms on medical devices such as a venous catheter or urinary catheter [10], [11]. This infection is greatly serious because biofilms are thought to be recalcitrant to antifungal (e.g., fluconazole) therapy [12], and only two classes of agents (i.e., amphotericin B and echinocandins) appear to have in vitro efficacy against *Candida* biofilms [13], [14].

A recent characterization of the “biofilm dispersal” phenomenon demonstrated that the dispersed cells have several virulence traits, distinct from planktonic cells [15]. Accordingly, formation of biofilm by *Candida* bloodstream isolates has been associated with increased virulence and mortality [16]–[19]. In this context, we previously showed that inadequate antifungal therapy, infection caused by biofilm-forming *Candida* isolates, and high Acute Physiology and Chronic Health Evaluation (APACHE) III score were independent predictors of mortality [18].

In the present study, we sought to determine risk factors for CBSI caused by biofilm-forming isolates and the impact of this infection on health and economic outcomes in adult patients.

## Methods

### Ethics Statement

The local institutional review committee approved the study, and informed consent was not required because of the observational nature of this study.

### Study Population and Design

We included all patients ≥18 years of age with culture-proven CBSI who were hospitalized during the period from 2005 through 2007 at the Catholic University Hospital in Rome, Italy, which is an academic tertiary care center with 1,500 beds and ∼50,000 hospital admissions per year. All patients were identified by electronically querying the clinical microbiology laboratory database. Some of these patients have been described elsewhere [20]. CBSI was defined as presence of ≥1 blood cultures growing *Candida* species. Only the first episode of CBSI was reported for patients with recurrent or subsequent episodes of infection. Patients whose cultures grew >1 documented species of *Candida* were excluded from analysis. Unless otherwise noted, the term “infection” refers to episode of candidemia under study.

This was a retrospective study consisting of two parts. For the case-case-control study, two groups of CBSI patients, one with infection caused by a biofilm-forming isolate and the other with infection caused by a non-biofilm-forming isolate, were compared with a common control group, consisting of randomly selected patients who had been hospitalized in our center during the same periods of time and in the same wards as the case patients, but who did not have evidence of CBSI [20]. Patients were included only if complete data series could be retrieved from their medical charts.

For the cohort study, patients with biofilm-forming CBSI were compared to those with non-biofilm-forming CBSI. Additionally, patients with biofilm-forming CBSI were matched to patients with non-biofilm-forming CBSI for the following factors: age (±10 years), sex, APACHE III score (±3), and receipt of adequate antifungal therapy [18]. If one patient could be matched to two or more patients, then the patient with the closest APACHE III score was selected.

### Variables and Definitions

Data were extracted from the patients' hospital records by using a standardized case report form and included demographics (age, sex); microbiological parameters (*Candida* species type, antifungal susceptibility profile, biofilm formation test result); comorbid conditions (diabetes mellitus, chronic obstructive pulmonary disease [COPD], chronic renal failure, solid organ cancer, hematologic malignancy, liver disease, human immunodeficiency virus [HIV] infection, neutropenia [absolute neutrophil count, <500 cells/mm^3^]); the Charlson's score was used as a composite index of comorbidities; invasive procedures (including insertion of a central venous catheter [CVC] or nasogastric tube, and urinary catheterization), and total parenteral nutrition (TPN) administration within 72 h prior to the onset of CBSI; use of immunosuppressive agents, surgery, bacteremia, or exposure to antibiotics within 30 days of the onset of candidemia (or, for controls, at any point during hospitalization) [20]; time at risk, i.e., number of hospital days from admission to the date of the first positive (index) blood culture for case patients or total days in the hospital for control patients [20]; antifungal therapy; and outcome parameters (hospital LOS following the onset of CBSI [post-CBSI hospital LOS], initial response to antifungal treatment, costs of antifungal therapy, hospital mortality [i.e., death within 30 days of the first documented CBSI episode], and infection-related mortality [i.e., mortality where the symptoms and signs of infection had not resolved at the time of death and there was no alternative cause of death]).

According to the European Center for Disease Prevention and Control guidelines [21], CBSI was defined as health care-associated if it occurred more than 48 h after admission to the hospital and if no signs or symptoms of infection were noted at the admission. For all cases, CBSI was considered to be catheter-related if quantitative roll-plate cultures of the catheter tip yielded more than 15 cfu of the same *Candida* species that was isolated from the bloodstream or if simultaneous quantitative cultures showed a ratio of ≥5∶1 in cfu of blood samples obtained through the catheter and a peripheral vein, or CBSI was considered to be catheter-associated if it occurred in a patient with an intravascular line in place at the time of, or within 48 h before, the onset of the infection [22]. The time of onset of CBSI was defined as the date in which the index culture for *Candida* was identified.

Adequate antifungal therapy was defined as the initiation of antifungal therapy given at a recommended dosage within 24 h after the index blood culture was obtained [23], with isolation of an organism that was found to be susceptible in vitro (see below) to the antifungal agent used.

### Microbiology

For *Candida* blood isolates, species identification was performed by micromorphology analysis and biochemical tests (Vitek 2 Yeast Identification, bioMérieux), whereas the antifungal susceptibility testing of planktonic cells was determined by broth microdilution method according to the Clinical and Laboratory Standards Institute M27-A3 document [24]. For the same isolates, biofilm formation was performed in batches using a known 96-well plate-based method [25], with slight modifications. Briefly, each well of polystyrene microtiter plates was inoculated with a *Candida* cell suspension consisting of 3×10^7^ cfu/mL in Sabouraud dextrose broth containing 8% glucose. After 24 h of incubation, planktonic cells were discarded by washing the wells three times manually with 0.15 M phosphate-buffered saline (PBS), and the remaining cells adherent to the plastic surface (biofilm) were quantified by i) a tetrazolium XTT [2,3-bis(2-methoxy-4-nitro-5-sulfophenyl)-2H-tetrazolium-5-carboxanilide] reduction assay and ii) direct absorbance measurement, as described previously [18]. In the first method, a 100-µl aliquot of the XTT salt solution (Sigma; 1 mg/mL in PBS) and 1 µM menadione solution (Sigma; prepared in acetone) were added to each prewashed biofilm and to control wells (for measurement of background XTT reduction levels). The plates were incubated in the dark at 37°C for 5 h, and the amount of XTT formazan was measured in a microtiter plate reader (microplate reader model 550; Bio-Rad) at 490 nm. Isolates for which the optical density was of <0.1 were scored as non-biofilm formers. In the second method, 200 µl of PBS was added to each well and biofilm was measured directly by spectrophotometric readings at 405 nm with the microtiter plate reader. The percent transmittance (%*T*) was calculated by subtracting the %*T* value for each test sample from the %*T* value for the reagent blank to obtain a measure of the amount of light blocked passing through the wells (%*T*_bloc_). On the basis of %*T*_bloc_ values, biofilm formation by each isolate was scored as either negative (%*T*_bloc_, <10) or positive (%*T*_bloc_, ≥10), according to which isolates were classified as non-biofilm formers or biofilm formers, respectively. *C. albicans* strain SC5314 was used as a control strain in each experiment.

### Statistical analysis

Following data collection, normally distributed continuous variables were reported as mean ± standard deviation (SD) and compared using Student's *t* test. Medians with ranges were used to describe nonnormally distributed continuous variables, and compared using the Mann-Whitney *U*-test. Categorical variables were reported as percentages and compared using the two-tailed χ^2^ test or Fisher's exact test, as appropriate. After univariate statistics were generated, all variables with a p-value of <0.20 were considered for inclusion in the multivariate logistic regression model to identify independent risk factors for the development of biofilm-related CBSI. We constructed a receiver operating curve (ROC) to assess the validity of the model. In the matched cohort study, comparisons of paired baseline characteristics were performed using the paired Student's *t* test and the McNemar test for continuous and categorical variables, respectively. The matched risk ratio and excess risk were expressed by ratio and difference in mortality rates between exposed and unexposed patients, respectively. Survival distribution function was estimated using the Kaplan-Meier product-limit method; nonparametric (log-rank and Wilcoxon) tests were used to compare the survival functions among the different groups. All p-values were two-tailed and statistical significance was defined as a p<0.05. Statistical analyses were performed using Stata, version IC 11.

## Results

During the study period, we identified a total of 222 case patients with CBSI. Fifteen patients, from whom blood cultures grew >1 species of *Candida*, were not included in the study. *C. albicans* was most commonly isolated (58.9%, 122 patients), whereas the majority of the other *Candida* species isolated included *C. parapsilosis* (22.7%, 47 patients), *Candida tropicalis* (9.6%, 20 patients), and *Candida glabrata* (5.3%, 11 patients). Other species (i.e., *Candida krusei*, *Candida lusitaniae*, and *Candida guilliermondii*) accounted for the remaining 3.4% of isolates (7 patients).

### Microbiological Findings

Eighty four (40.5%) of 207 patients were infected by biofilm-forming *Candida* isolates, of which 32 were *C. albicans*, 29 were *C. parapsilosis*, 13 were *C. tropicalis*, 7 were *C. glabrata*, 2 were *C. krusei*, and 1 was *C. guilliermondii*, as assessed by the XTT and %*T* assays (see Methods). The levels of biofilm formation quantified by the XTT reduction assay for the 84 *Candida* isolates ranged from 0.125 to 1.358 (median, 0.592), whereas the corresponding levels determined by the %*T* assay ranged from 11 to 62 (median, 26) (Table S1). Overall, biofilm production by *C. albicans* was significantly less frequent (26.2%, 32 of 122 isolates) than non-*C. albicans* species (61.1%, 52 of 85 isolates) (*P*<0.001). Among the latter species, biofilm production was most frequently observed for *C. tropicalis* (70.0%, 14 of 20 isolates), followed by *C. glabrata* (63.6%, 7 of 11 isolates), and *C. parapsilosis* (61.7%, 29 of 47 isolates). All planktonically growing isolates were found to be susceptible in vitro to amphotericin B, flucytosine, caspofungin, and anidulafungin, and, except for fluconazole-resistant *C. glabrata* (3 isolates) and *C. krusei* isolates, to azoles.

### Patients Characteristics

The demographic and clinical characteristics of case (*n* = 207) and control (*n* = 200) patients are shown in Table 1. At the time of candidemia, 36 (17.4%) patients were in the ICU and 84 (40.6%) patients had a surgical admission. In spite of 141 (68.1%) catheter-associated infections, there were 68 (32.8%) CVC-related candidemia cases (diagnosed by catheter cultures in 37 cases and by differential quantitative blood cultures of ≥5∶1 ratio in 31 cases). The primary source of infection was unknown for 96 (46.4%) patients. Of case patients, 170 (82.1%) received adequate systemic antifungal therapy, with a median duration of 24 days (range, 2–46 days). Fluconazole was most frequently used (96 patients, 56.5%), followed by caspofungin (41 patients, 24.1%), lipid formulations of amphotericin B (21 patients, 12.3%), and voriconazole (12 patients, 7.1%). In particular, azoles (mostly fluconazole) were administered to 47 (55.9%) of 84 patients with biofilm-forming CBSI, and to 49 (39.8%) of 123 patients with non-biofilm-forming CBSI. Therapy was considered inadequate for 37 (17.9%) patients. Of these, 6 patients (fluconazole-treated) were infected by fluconazole-resistant *C. glabrata* or *C. krusei* isolates; 31 patients received antifungal treatment after 48 h of the time that the index blood culture was obtained. Catheter removal was part of treatment in 129 (91.5%) of the 141 patients with catheter-associated CBSI, and in 67 (98.5%) of the 68 patients with catheter-related CBSI.

**Table 1:** Univariate analysis of risk factors for candidemias by biofilm-forming (BF) and non-biofilm-forming (NBF) isolates.

Variable	Control group (*n* = 200)	BF-CBSI group (*n* = 84)	BF-CBSI group vs. control group		NBF-CBSI group (*n* = 123)	NBF-CBSI group vs. control group	
			OR (95% CI)	P-value		OR (95% CI)	P-value
Demographic parameters							
Age, yrs	66 (22–97)	65.5 (15–91)	-	0.79	64 (18–99)	-	0.52
Male sex	106 (53.0)	47 (55.9)	1.12 (0.65–1.94)	0.65	68 (55.3)	1.09 (0.68–1.76)	0.69
Hospital LOS, median (range) days^a^	14 (2–87)	41 (11–277)	-	<0.001	42 (4–207)	-	<0.001
Time at risk, median (range) days^b^	14 (2–87)	18.5 (3–192)	-	0.05	19 (2–157)	-	0.01
Neutropenia^c^	0	6 (7.1)	-	<0.001	4 (3.3)	-	0.01
Comorbid condition							
COPD	6 (3.0)	17 (20.2)	8.20 (2.91–26.25)	<0.001	23 (18.7)	7.43 (2.80–22.91)	<0.001
Solid organ cancer	54 (27.0)	34 (40.5)	1.83 (1.03–3.24)	0.02	50 (40.6)	1.85 (1.11–3.06)	0.01
Hematologic cancer	7 (3.5)	8 (9.5)	2.90 (0.88–9.71)	0.03	9 (7.3)	2.17 (0.69–7.06)	0.12
Diabetes mellitus	31 (15.5)	34 (40.5)	3.70 (1.98–6.89)	<0.001	15 (12.2)	0.75 (0.36–1.53)	0.41
Chronic renal failure	32 (16.0)	21 (25.0)	1.75 (0.88–3.39)	0.07	36 (29.3)	2.17 (1.21–3.87)	0.004
Liver disease	14 (7.0)	9 (10.7)	1.59 (0.58–4.14)	0.29	12 (9.8)	1.43 (0.58–3.47)	0.37
HIV infection	0	1 (1.2)	-	0.12	3 (2.4)	-	0.02
Charlson's score median (range)	2 (0–8)	3 (0–15)	-	<0.001	3 (0–15)	-	<0.001
Invasive procedures							
CVC	33 (16.5)	56 (66.6)	10.12 (5.40–19.02)	<0.001	85 (69.1)	11.31 (6.41–20.04)	<0.001
Nasogastric tube	30 (15.0)	27 (32.1)	2.68 (1.40–5.10)	0.001	44 (35.8)	3.15 (1.78–5.59)	<0.001
Urinary catheter	65 (32.5)	64 (76.2)	6.64 (3.58–12.54)	<0.001	73 (59.4)	3.03 (1.85–4.96)	<0.001
Total parenteral nutrition	38 (19.0)	58 (69.1)	9.51 (5.11–17.78)	<0.001	85 (69.1)	9.53 (5.49–16.61)	<0.001
Previous bacteremia^d^	5 (2.5)	23 (27.4)	14.70 (5.11–51.06)	<0.001	32 (26.0)	13.71 (5.03–46.19)	<0.001
Previous surgery	48 (24.0)	38 (45.2)	2.61 (1.47–4.63)	<0.001	67 (54.5)	3.78 (2.27–6.31)	<0.001
Prior use of:							
corticosteroids	38 (19.0)	29 (34.5)	2.24 (1.21–4.14)	0.05	45 (36.6)	2.45 (1.43–4.22)	<0.001
immunosuppressive agents	22 (11.0)	16 (19.1)	1.90 (0.87–4.04)	0.07	24 (19.5)	1.96 (0.99–3.86)	0.03
broad-spectrum antibiotics	115 (57.5)	75 (89.3)	6.15 (2.85–14.70)	<0.001	115 (93.5)	10.62 (4.82–26.40)	<0.001

**NOTE.** Results are shown as no. (%) or median with range. LOS, length of stay; CBSI, *Candida* bloodstream infection; COPD, chronic obstructive pulmonary disease; CVC, central venous catheter; HIV, human immunodeficiency virus.

a Days of hospital stay prior to the onset of candidemia (or, for controls, total days in the hospital).

b Number of hospital days from admission to the onset of candidemia (or, for controls, total days in the hospital).

c Absolute neutrophil count <500 cells/mm^3^.

d Within 30 days prior to the onset of candidemia (or, for controls, at any point during hospitalization).

### Univariate and Multivariate Analyses

The 84 patients with biofilm-forming CBSI and 123 patients with non-biofilm-forming CBSI were more likely than control patients to have longer time at risk (p = 0.05 and p = 0.01, respectively) or hospital LOS (p<0.001), underlying condition such as COPD (p<0.001) or solid organ cancer (p = 0.02 and p = 0.01, respectively), or higher Charlson's score (p<0.001); they were more likely to have central venous catheterization (p<0.001), urinary catheterization (p<0.001), total parenteral nutrition (p<0.001), neutropenia (p<0.001 and p = 0.01), prior bacteremia (p<0.001), or previous exposure to surgery (p<0.001), corticosteroids (p = 0.005 and p<0.001, respectively), or broad-spectrum antibiotics (p<0.001) (Table 1). Patients with biofilm-forming CBSI were more likely to have diabetes mellitus (p<0.001) or hematologic malignancy (p = 0.03), whereas patients with non-biofilm-forming CBSI were more likely to have chronic renal failure (p = 0.004), HIV infection (p = 0.02), or previous exposure to immunosuppressive agents (p = 0.03) (Table 1).

Table 2 displays the results from the multivariate logistic regression analysis. Central venous catheter use (OR 6.44; 95% CI 3.21–12.92), total parenteral nutrition administration (OR 5.21; 95% CI 2.59–10.48), diabetes mellitus (OR 4.47; 95% CI 2.03–9.83), and urinary catheter use (OR 2.40; 95% CI 1.18–4.91) were significantly associated with CBSI caused by a biofilm-forming isolate. Together with the administration of total parenteral nutrition (OR 8.41; 95% CI 3.70–19.08) or the presence of a central venous catheter (OR 5.73; 95% CI 2.55–12.84), prior broad-spectrum antibiotic use (OR 4.48; 95% CI 1.55–12.93) and previous surgery (OR 2.45; 95% CI 1.04–5.81) were instead independent risk factors for CBSI caused by a non-biofilm-forming isolate. The ROC AUCs for the two multivariate models were 0.96 and 0.95 for biofilm-forming and non-biofilm-forming CBSI, respectively, indicating that the models have excellent predictive power.

**Table 2:** Logistic regression analysis of risk factors for candidemias by biofilm-forming (BF) and non-biofilm-forming (NBF) isolates.

Variable	OR (95% CI)
**BF CBSI**	
CVC in place at time of positive blood culture	6.44 (3.21–12.92)
Total parenteral nutrition	5.21 (2.59–10.48)
Diabetes mellitus	4.47 (2.03–9.83)
Urinary catheter in place at time of positive blood culture	2.40 (1.18–4.91)
**NBF CBSI**	
Total parenteral nutrition	8.41 (3.70–19.08)
CVC in place at time of positive blood culture	5.73 (2.55–12.84)
Antibiotic therapy in previous 30 days	4.48 (1.55–12.93)
Surgery in previous 30 days	2.45 (1.04–5.81)

**NOTE.** CBSI, *Candida* bloodstream infection; CVC, central venous catheter.

### Outcomes

**Figure 1:**
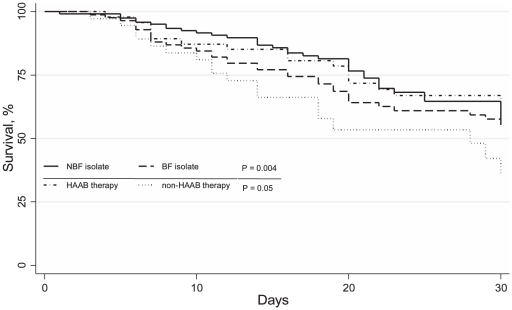
Survival among patients with *Candida* bloodstream infection (CBSI) at 30 days. Patients were grouped according to the biofilm-forming (BF) or non-biofilm-forming (NBF) *Candida* isolate (for all CBSIs), and according to receiving of highly active anti-biofilm (HAAB) or non-HAAB antifungal therapy (for BF CBSIs only). P-values for statistically significant differences between the groups are shown.

**Figure 2:**
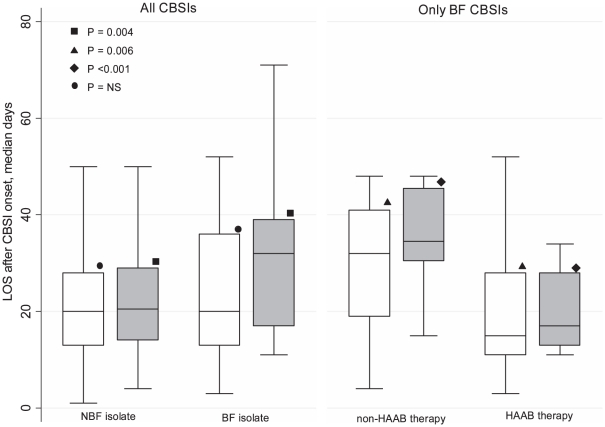
Hospital length of stay (LOS) following the *Candida* bloodstream infection (CBSI) onset in all (white box-plots) or surviving (grey box-plots) patients. Patients were grouped according to the biofilm-forming (BF) or non-biofilm-forming (NBF) *Candida* isolate (for all CBSIs), and according to receiving of highly active anti-biofilm (HAAB) or non-HAAB antifungal therapy (for BF CBSIs only). Lines inside the boxes indicate the median values, whereas upper and lower limits of the boxes and whiskers indicate the interquartile and total ranges, respectively. P-values for statistically significant differences between the groups are shown.

The hospital mortality was 51.2% (43 of 84 patients) in the biofilm-forming CBSI group, compared with 31.7% (39 of 123 patients) in the non-biofilm-forming CBSI group (p = 0.004), with infection-related mortality rates of 44.1% (37 of 84 patients) and 27.6% (34 of 123 patients), respectively (p = 0.01). Differences in the 30-day survival distributions were found between the two groups by overall (p = 0.004) (Figure 1), and when the biofilm-forming CBSI group was stratified by therapy with highly active anti-biofilm (HAAB; e.g., caspofungin) or non-HAAB (e.g., fluconazole) antifungal agents (p = 0.05) (Figure 1). As shown in Figure 2, the median (range) post-CBSI hospital LOS did not significantly differ between biofilm-forming CBSI group (20 days [3–189]) and non-biofilm-forming CBSI group (19 days [1–105]) (p = 0.16), by considering all case patients; however, this difference between the groups reached statistical significance when calculated only among patients who survived (32 days [11–189] vs. 20.5 days [4–105], respectively, p = 0.004). Among patients with biofilm-forming CBSI, the median post-CBSI hospital LOS was longer (32 [4–189] days) in the group receiving non-HAAB antifungal therapy compared to the HAAB antifungal therapy group (15 [3–85] days) (p = 0.006); also by considering only the survivors, patients receiving non-HAAB antifungal therapy had longer post-CBSI hospital LOS than those receiving HAAB antifungal therapy (33.5 [14–188] days vs. 16 [10–89] days, p<0.001) (Figure 2). As expected, the mean antifungal therapy cost was higher for patients with biofilm-forming CBSI than those with non-biofilm-forming CBSI (€ 10,804±6590 vs. 6067±3934, p = 0.04).

### Results of a Matched Cohort Study

Seventy three (86.9%) of 84 patients with CBSI caused by a biofilm-forming isolate could be matched to 73 patients with CBSI caused by a non-biofilm-forming isolate, based on age, sex, APACHE III score, and adequateness of antifungal therapy. A comparison of patient characteristics between the two groups is presented in Table 3. Similarly to that presented above, significantly higher hospital mortality, post-CBSI hospital LOS, and antifungal therapy costs were observed in biofilm-forming CBSI patients compared to non-biofilm-forming CBSI patients (p = 0.004, p = 0.007, and p = 0.02, respectively). Furthermore, the biofilm-related infection was associated with a matched excess risk for death in hospital of 23.3% (53.4% vs. 30.1%, p = 0.004), and the matched risk ratio was 1.77.

**Table 3:** Comparison between patients with biofilm-forming (BF) candidemia or non-biofilm-forming (NBF) candidemia in the matched cohort study.

Variable	BF-CBSI group (*n* = 73)	NBF-CBSI group (*n* = 73)	P-value
Hospital LOS, days^a^	40 (11–277)	35 (15–150)	0.21
ICU stay at diagnosis	9 (12.3)	11 (15.1)	0.63
Time at risk, days^b^	20 (3–192)	15 (2–123)	0.41
Species isolated			
*Candida albicans*	30 (41.1)	52 (71.2)	<0.001
*Candida parapsilosis*	24 (32.9)	10 (13.7)	0.006
*Candida tropicalis*	10 (13.7)	5 (6.8)	0.17
*Candida glabrata*	6 (8.2)	3 (4.1)	0.30
Other^c^	3 (4.1)	3 (4.1)	1.00
Neutropenia^d^	6 (8.2)	4 (5.5)	0.51
Comorbid conditions			
COPD	16 (21.9)	16 (21.9)	1.00
Solid organ cancer	29 (39.7)	28 (38.3)	0.86
Hematologic cancer	7 (9.6)	6 (8.2)	0.77
Diabetes mellitus	28 (38.4)	10 (13.7)	<0.001
Chronic renal failure	17 (23.3)	18 (24.6)	0.84
Liver disease	9 (12.3)	7 (9.6)	0.59
HIV infection	1 (1.4)	1 (1.4)	1.00
Charlson's score	3 (0–15)	3 (0–10)	0.58
Invasive procedures			
CVC	48 (65.7)	52 (71.2)	0.47
Nasogastric tube	24 (32.9)	23 (31.5)	0.85
Urinary catheter	57 (78.1)	38 (52.1)	<0.001
Total parenteral nutrition	51 (69.9)	55 (75.3)	0.45
Previous bacteremia^e^	20 (27.4)	18 (24.6)	0.70
Previous surgery	32 (43.8)	38 (52.1)	0.32
Prior use of:			
corticosteroids	23 (31.5)	28 (38.3)	0.38
immunosuppressive agents	15 (20.5)	18 (24.6)	0.55
broad-spectrum antibiotics	66 (90.4)	67 (91.8)	0.77
Outcome parameters			
Initial treatment failure^f^	26 (35.6)	15 (20.5)	0.04
Hospital LOS after CBSI, days	29±31	19±5	0.007
Hospital mortality	39 (53.4)	22 (30.1)	0.004
Infection-related mortality	32 (43.8)	18 (24.6)	0.01
Antifungal therapy cost	€ 11,371±6544	€ 6108±4106	0.02

**NOTE.** Results are shown as no. (%) or median with range. LOS, length of stay; ICU, intensive care unit; CBSI, *Candida* bloodstream infection; COPD, chronic obstructive pulmonary disease; HIV, human immunodeficiency virus; CVC, central venous catheter.

a Days of hospital stay prior to the onset of candidemia (or, for controls, total days in the hospital).

b Number of hospital days from admission to the onset of candidemia (or, for controls, total days in the hospital).

c Other species includes *Candida krusei* (3 cases), *Candida lusitaniae* (2 cases), and *Candida guilliermondii* (1 case).

d Absolute neutrophil count <500 cells/mm^3^.

e Within 30 days prior to the onset of candidemia (or, for controls, at any point during hospitalization).

f Therapeutic failure at 72 h after starting antifungal therapy, as assessed by the persistence of infection or by the occurrence of death.

## Discussion

In the present study, we identified two unique risk factors, diabetes mellitus and urinary catheterization, that were specifically associated with biofilm-forming CBSI. Diabetes mellitus has previously been reported to be a general risk factor for *Candida* infections [10]. Yet, glucose is thought to serve as the carbohydrate energy source required by *Candida* for biofilm formation [26], perhaps necessary to produce the polysaccharide matrix [27], in which organized communities of yeast, hyphae, and pseudohyphae are enclosed [11]. Hence, it is plausible that a hyperglycemic condition may favor adaptation of *Candida* organisms to a biofilm lifestyle, and this should be consistent with enhanced pathogenic potential of biofilm-forming *C. albicans* strains isolated from patients with type 1 diabetes [28]. Catheter-associated urinary tract infections result in increased institutional death rates [29], and are frequently associated with the formation of biofilms on the catheter surfaces [10], [30]. Whilst *Candida* biofilms in vitro are formed frequently in nutrient-rich laboratory media (i.e., containing up to 8% of dextrose [16], [18]), it was yet shown that a synthetic urine medium (i.e., use of an in vitro model mimicking an in vivo biofilm on a urinary catheter) was able to support the biofilm growth of *Candida* [31], making verisimilar the likelihood that fungal cells may spread from the infected urinary catheter and seed the bloodstream, thereby promoting systemic candidiasis [32].

A wide range of biomaterials used in clinical practice are shown to support colonization and biofilm formation by *Candida* species [30], making device-related *Candida* infections relatively refractory to medical therapy [10]. It has been reported that certain *Candida* species in the presence of glucose-containing fluids or lipid emulsion might produce “slime” (now commonly referred to as biofilm), potentially explaining the increased proportion of CBSIs among patients receiving parenteral nutrition [16], [22], [26]. Surprisingly, we found that use of CVC and receipt of total parenteral nutrition were independently associated with all CBSIs in our patients. A possible explanation for this finding is that not all of our candidemia episodes originated from a catheter, as ∼50% of CBSIs had unrecognized primary sources. Of note, 59 of 139 patients with no CVC-related CBSI were infected by biofilm-forming isolates, supporting the notion that the adherence properties of infecting organisms are important, but not the sole pathogenic determinants of catheter-related *Candida* infection [22]. In addition, the finding that either biofilm producers (25 patients) or non-biofilm producers (43 patients) were found in our patients who have a CVC-related candidemia makes difficult to determine the role played by biofilms in the pathogenesis of this infection. As removal of CVC regarded almost the totality of our patients, it was not possible to establish whether patients with biofilm-forming CBSI benefited from this therapeutic decision more than patients with non-biofilm-forming CBSI. Consistently, it also seems likely that non-device-related infections, such as certain infections on epithelial surfaces involve *Candida* biofilms [11].

Nonetheless, our main goal in this study was to evaluate the effect of biofilm production on clinical (mortality and length of stay) and economic (costs of antifungal therapy) outcomes of patients with CBSI, in an attempt to support and extend (different years are included in the present analysis) our previous findings that, together with well-established factors (i.e., inadequate antifungal therapy and high APACHE III score), infection with overall biofilm-forming *Candida* species is an independent predictor of mortality for patients with candidemia [18]. In particular, we previously found that only infections caused by *C. albicans* and *Candida parapsilosis* were associated with higher mortality rates, leading us to state that the capability to form biofilms may be an important contributor to the virulence of *C. albicans* or enhance the pathogenic potential of *C. parapsilosis* [18].

Here, we initially determined the attributable adverse effects of biofilm formation on patient outcomes, by analyzing patients with biofilm-forming CBSI who were substantially different from the patients with non-biofilm-forming CBSI. Hence, to partially eliminate the effects of intergroup differences on patient outcomes, we have chosen to compare groups of matched patients who were very similar for age, sex, APACHE III score, and receipt of adequate antifungal treatment, but differed regarding the other variable(s) of interest. We found that not only hospital mortality but also post-CBSI hospital LOS and costs of antifungal therapy were significantly greater among patients infected by biofilm-forming isolates than those of patients infected by non-biofilm-forming isolates. Thus, we assessed (in absolute novelty with respect to our previous study) whether the treatment with a HAAB antifungal agent might significantly affect all the outcomes we analyzed. In this context, a recent work suggested that the total treatment cost of candidemia is strongly influenced by the choice of initial antifungal treatment, that increases overall expenditure largely creating a need for additional hospital days [33]. In our study, 55.9% of patients treated for biofilm-forming CBSI were treated with azole antifungals. As Figure 2 shows, the post-CBSI hospital LOS was significantly longer in patients who received non-HAAB antifungal therapy (e.g., fluconazole [34]), compared to patients treated with a HAAB antifungal agent (e.g., echinocandins [14], [34]). Noteworthy, by considering only patients who survived, this difference favored again significantly the HAAB antifungal treatment (Figure 2). As the choice of first-line antifungal agent should take into account the actual possibility of a biofilm-forming CBSI, our findings add support to the growing evidence that echinocandin drugs may effectively act against biofilm-related infections [35], [36]. The management of biofilm-forming *Candida* infections is greatly influenced by their persistent nature and associated drug resistance [12], perhaps because of the dispersion of “persister” antifungal-resistant biofilm cells [15]. It is plausible that the excess of mortality we observed in our matched patients could be attributed to the difficulty to prevent complete eradication of organisms from the blood or to eliminate a potential nidus of infection that may perpetuate seeding of the bloodstream.

The main limitations of the present study were the retrospective design which may have predisposed it to a selection bias, and the single-center nature which may limit the results' generalization to other centers.

In conclusion, our data expand current knowledge of the impact of hospital-acquired CBSI on the patients' survival, and point out the major risk factors for biofilm-related candidemia. Also, we shed light on the potential cost and hospital resource savings that may be possible if biofilm-forming CBSIs would be promptly treated with more effective antifungal agents.

## Supporting Information

Table S1 **Biofilm formation by 84** ***Candida*** **isolates from candidemic patients as assayed by two quantitative methods.**
(DOC)
