# Supplementary material for: Risk Factors and Outcomes of Candidemia Caused by Biofilm-Forming Isolates in a Tertiary Care Hospital
Source: PLoS One. 2012 Mar 30;7(3):e33705. doi: 10.1371/journal.pone.0033705 (PMC3316499; doi:10.1371/journal.pone.0033705)
Supplement: Table S1 — Biofilm formation by 84 Candida isolates from candidemic patients as assayed by two quantitative methods. (DOC) [file pone.0033705.s001.doc]

**Table S1**. Biofilm formation by 84 *Candida* isolates from candidemic patients as assayed by two quantitative methods.

| No. of isolate | Species of *Candida* | Testing assay: | |
| --- | --- | --- | --- |
|  |  | XTT | %T_bloc_ |
| C1  C2  C4  C5  C9  C12  C14  C15  C19  C21  C24  C26  C27  C29  C30  C32  C35  C37  C38  C39  C42  C43  C46  C48  C50  C55  C56  C58  C61  C63  C64  C65  C69  C70  C72  C73  C79  C82  C85  C88  C92  C94  C95  C99  C100  C103  C109  C110  C111  C112  C115  C117  C119  C120  C125  C127  C128  C132  C135  C138  C140  C142  C144  C149  C152  C153  C159  C161  C164  C165  C171  C172  C175  C179  C181  C185  C189  C190  C191  C194  C198  C200  C201  C205 | *C. albicans*  *C. parapsilosis*  *C. tropicalis*  *C. albicans*  *C. tropicalis*  *C. albicans*  *C. parapsilosis*  *C. albicans*  *C. parapsilosis*  *C. parapsilosis*  *C. albicans*  *C. albicans*  *C. albicans*  *C. parapsilosis*  *C. albicans*  *C. tropicalis*  *C. albicans*  *C. parapsilosis*  *C. parapsilosis*  *C. albicans*  *C. krusei*  *C. tropicalis*  *C. parapsilosis*  *C. albicans*  *C. tropicalis*  *C. parapsilosis*  *C. albicans*  *C. albicans*  *C. albicans*  *C. albicans*  *C. tropicalis*  *C. parapsilosis*  *C. albicans*  *C. parapsilosis*  *C. albicans*  *C. glabrata*  *C. parapsilosis*  *C. albicans*  *C. albicans*  *C. albicans*  *C. parapsilosis*  *C. parapsilosis*  *C. albicans*  *C. parapsilosis*  *C. parapsilosis*  *C. albicans*  *C. parapsilosis*  *C. tropicalis*  *C. tropicalis*  *C. albicans*  *C. parapsilosis*  *C. parapsilosis*  *C. glabrata*  *C. albicans*  *C. parapsilosis*  *C. albicans*  *C. parapsilosis*  *C. tropicalis*  *C. albicans*  *C. parapsilosis*  *C. albicans*  *C. parapsilosis*  *C. glabrata*  *C. tropicalis*  *C. albicans*  *C. parapsilosis*  *C. glabrata*  *C. albicans*  *C. parapsilosis*  *C. parapsilosis*  *C. albicans*  *C. guilliermondii*  *C. albicans*  *C. glabrata*  *C. tropicalis*  *C. albicans*  *C. krusei*  *C. parapsilosis*  *C. glabrata*  *C. tropicalis*  *C. parapsilosis*  *C. glabrata*  *C. tropicalis*  *C. parapsilosis* | 0.125  0.582  1.125  0.238  1.026  0.596  0.835  0.856  0.685  0.312  0.596  0.305  0.452  0.595  0.623  0.963  0.198  0.458  0.325  0.251  0.623  1.230  0.623  0.723  0.853  0.147  0.497  0.605  0.238  0.589  1.156  0.825  0.402  0.256  0.562  0.236  0.423  0.691  0.521  0.420  0.702  0.780  0.485  0.320  0.160  0.509  0.758  0.940  0.896  0.450  0.206  0.580  0.359  0.642  0.560  0.180  0.805  1.156  0.125  0.595  0.205  0.575  0.685  1.145  0.633  0.841  0.720  0.283  0.785  0.605  0.441  0.556  0.153  0.621  0.974  0.296  0.792  0.193  0.585  1.358  0.680  0.452  1.203  0.598 | 12  25  58  16  45  27  35  31  34  19  28  16  19  26  29  49  15  25  17  15  30  62  32  35  40  11  22  28  16  27  51  42  19  16  22  14  18  29  22  18  31  35  21  15  12  23  34  48  39  21  16  26  19  30  23  13  32  54  12  25  15  24  33  50  31  40  34  18  38  27  20  23  14  28  42  18  36  14  26  62  32  21  53  25 |
